# Late-Onset Posttransplant Lymphoproliferative Disorder Results in Jejunal Stricture Managed with Endoscopic Dilation

**DOI:** 10.1155/2021/5583665

**Published:** 2021-08-03

**Authors:** Steven Park, John Miller, Janice Cheong, Asad Ullah, Lawrence Saubermann, Arthur Decross

**Affiliations:** ^1^Department of Internal Medicine, University of Rochester Medical Center, Rochester, NY, USA; ^2^Division of Gastroenterology & Hepatology, University of Rochester Medical Center, Rochester, NY, USA

## Abstract

**Background:**

Late-onset posttransplant lymphoproliferative disorder (PTLD) after orthotopic heart transplantation is rare. *Case Presentation*. We present a rare diagnosis of small bowel stricture caused by healed lymphomatous ulcers in a patient with orthotopic heart transplantation and PTLD diagnosed 25 years after initial transplantation. We also demonstrate successful endoscopic balloon dilations that improved the patient's obstructive symptoms.

**Conclusion:**

It is important to consider stricture from healed lymphomatous ulcers in posttransplant patients presenting with obstructive symptoms.

## 1. Background

Late-onset posttransplant lymphoproliferative disorder (PTLD) after orthotopic heart transplantation is rare. We present a rare diagnosis of small bowel stricture caused by healed lymphomatous ulcers in a patient with orthotopic heart transplantation and PTLD diagnosed 25 years after initial transplantation. We also demonstrate successful endoscopic balloon dilations that improved the patient's obstructive symptoms.

## 2. Case Presentation

A 31-year-old female with a past medical history of two orthotopic heart transplants (at age six for viral cardiomyopathy and at age thirteen for graft vasculopathy) complicated by posttransplant lymphoproliferative disorder (PTLD) (monomorphic with germinal center B-cell type; EBV-negative) presented to our hospital with two weeks of postprandial emesis. Nausea and emesis occurred two to four hours after meals and consisted of nonbloody, nonbilious emesis. Bloodwork was notable for hemoglobin of 8 g/dL (11.2–15.7 g/dL). 6 days prior to admission, she had received third cycle of triweekly chemotherapy with cyclophosphamide, doxorubicin, vincristine, and prednisone (CHOP).

Upper endoscopy performed 1 month prior to admission to evaluate for a source of anemia demonstrated scattered, large, nonbleeding, clean-based ulcers in the proximal jejunum and a segment of circumferential ulceration. Biopsies of the jejunal ulcers confirmed involvement by patient's known monomorphic-type PTLD (germinal center B-cell type; EBV-negative). To evaluate for gastric outlet obstruction (GOO) suggested by CT scan in the setting of nausea and emesis, we performed an upper endoscopy. This endoscopy revealed a wide-open pylorus without GOO, and no endoscopic abnormality was seen through the fourth portion of the duodenum. Duodenal biopsies were negative for cytomegalovirus, adenovirus, and herpes simplex. Congo red stains were negative for amyloid, and morphologic evidence of lymphoma was absent.

**Figure 1:**
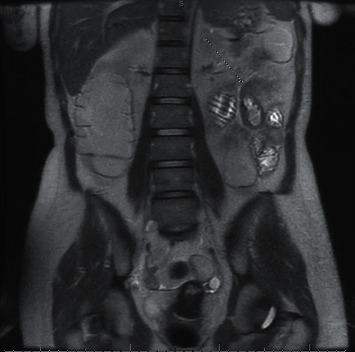
MR enterography showing small bowel obstruction with a transition point at focal thickening of the proximal jejunum (arrow).

**Figure 2:**
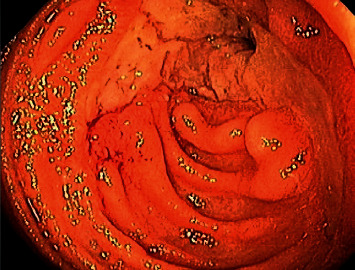
EGD showing a clean-based circumferential ulceration in the proximal jejunum with an ulcerated stricture estimated 2 mm in luminal diameter.

We were suspicious of a more distal site of obstruction and ordered an MR enterography (Figure 1) that revealed a transition point with focal thickening of the proximal jejunum. The decision was made to pursue endoscopic balloon dilation. On push enteroscopy, we encountered an ulcerated stricture with only 2-3 mm in luminal diameter (Figure 2). The site of stricture appeared to be the same segment of jejunal ulceration on initial enteroscopy that likely healed with scarring after chemotherapy. Decision was made to proceed with endoscopic dilation using a 6 mm biliary dilation balloon catheter, the smallest dilation catheter available via long pediatric colonoscope instrument channel. Roughly 4 mm diameter distention was reached before notable resistance was felt and evidence of trauma was seen. Patient tolerated a full liquid diet postprocedure and was discharged with a plan for a repeat dilation. To date, patient completed two additional endoscopic dilation procedures, followed by a small bowel resection, and remains alive after completion of total 6 rounds of R-CHOP without additional gastrointestinal complications.

## 3. Discussion and Conclusions

PTLD is a heterogeneous lymphoid proliferation that occurs after solid-organ or hematopoietic transplantation in the setting of prolonged immunosuppression [1–3]. The median time to diagnosis after transplantation is 2.5 years, and the incidence of PTLD in cardiac transplant patients ranges from 5 to 9% [4–6]. The clinical manifestations vary from nonspecific symptoms to sudden enlargement of nodal and extranodal lymphoid organs [7]. Lymphoid proliferations range from benign collections to malignant lymphomas involving T cells, B cells, and/or plasma cells [8]. PTLD is categorized based on the WHO classification of lymphoid neoplasm, and three main stages of evolution are recognized: early, polymorphic, and monomorphic lesions [9]. Polymorphic and monomorphic B-cell PTLD are the two most common histologic subsets, with increasing proportion of monomorphic cases noted in recent decades [8]. Of note, our patient had monomorphic-type PTLD with negative EBV stain, germinal center B-cell type with immunohistochemistry negative for NYC and positive for BCL2.

PTLD can also involve other organs such as central nervous system, bone marrow, spleen, lung, liver, and kidney [7]. PTLD involvement of small bowel in the form of GI bleed and viral infection has been reported previously [10–13]. However, a stricture in the small bowel from healed lymphomatous ulcer causing obstruction and needing endoscopic dilation has not been reported to our knowledge.

Our case highlights a rare diagnosis of small bowel stricture caused by healed lymphomatous ulcers in a patient with orthotopic heart transplantation and PTLD diagnosed 25 years after initial transplantation. We demonstrate successful endoscopic balloon dilations that improved the patient's obstructive symptoms. A multidisciplinary care involving oncology, pathology, gastroenterology, and colorectal surgery service was crucial to addressing gastrointestinal complications of PTLD. It is important to consider stricture from healed lymphomatous ulcers in posttransplant patients presenting with obstructive symptoms.

## Data Availability

No data were used to support this study.

## Abbreviations

PTLD: Posttransplant lymphoproliferative disorder
CHOP: Cyclophosphamide, doxorubicin, vincristine, and prednisone
GOO: Gastric outlet obstruction
MR: Magnetic resonance.
